# Poly(3,4-ethylenedioxythiophene) Based Solid-State Polymer Supercapacitor with Ionic Liquid Gel Polymer Electrolyte

**DOI:** 10.3390/polym12020297

**Published:** 2020-02-02

**Authors:** Haiyan Du, Zemin Wu, Yuyu Xu, Shaoze Liu, Huimin Yang

**Affiliations:** College of Chemistry and Chemical Engineering, Taiyuan University of Technology, Yingze West Street 79, Taiyuan 030024, China; wuzm101128@163.com (Z.W.); xuyuyu@tyut.edu.cn (Y.X.); liushaoze@tyut.edu.cn (S.L.);

**Keywords:** poly(3,4-ethylenedioxythiophene), 1-butyl-3-methylimidazolium tetrafluoroborate, gel polymer electrolyte, solid state supercapacitor

## Abstract

In this work, solid-state polymer supercapacitor (SSC) was assembled using poly(3,4-ethylenedioxythiophene/carbon paper (PEDOT/CP) as an electrode and ionic liquid (1-butyl-3-methylimidazole tetrafluoroborate)/polyvinyl alcohol/sulfuric acid (IL/PVA/H_2_SO_4_) as a gel polymer electrolyte (GPE). The GPE was treated through freezing–thawing (F/T) cycles to improve the electrochemical properties of PEDOT SSC. Cyclic voltammetry (CV), galvanostatic charge–discharge measurements (GCD) and electrochemical impedance spectroscopy (EIS) techniques and conductivity were carried out to study the electrochemical performance. The results showed that the SSC based on ionic liquid GPE (SSC-IL/PVA/H_2_SO_4_) has a higher specific capacitance (with the value of 86.81 F/g at 1 mA/cm^2^) than the SSC-PVA/H_2_SO_4_.The number of F/T cycles has a great effect on the electrochemical performance of the device. The energy density of the SSC treated with 3 F/T cycles was significantly improved, reaching 176.90 Wh/kg. Compared with the traditional electrolytes, IL GPE has the advantages of high ionic conductivity, less volatility, non-flammability and wider potential window. Moreover, the IL GPE has excellent elastic recovery and self-healing performance, leading to its great potential applications in flexible or smart energy storage equipment.

## 1. Introduction

Recently, electrochemical energy storage devices have received much attention due to the increasing population and the sharp depletion of fossil fuels [1,2,3,4]. Supercapacitors (SCs) are a promising green energy storage device because of their high power density, fast charge–discharge rate, long-cycle stability, and eco-friendliness [5,6,7,8,9,10]. These attractive properties have prompted their use in electric vehicles, wearable electronic and medical electronic devices, and military equipment [11,12,13].

Electrolyte and electrode materials are the important components of SCs. Different types of electrolytes, including liquid, solid state electrolytes, and gel polymer electrolyte, have been widely used for SCs. Compared with the conventional liquid electrolyte, solid-state polymer electrolytes (SPEs) and gel polymer electrolytes (GPEs) have the advantages of avoiding liquid leakage and corrosion problems, having a simple packaging and manufacturing process, good electrochemical stability, etc. [14,15,16]. However, SPEs are restrictively applied, due to their low conductivity and poor elastic or flexible properties, which limit the applications of SPEs in flexible or stretchable devices. GPEs are basically a liquid electrolyte (an ionic salt dissolved into an organic solvent or ionic liquids (ILs)) trapped inside a polymer network, which possess both the properties of solids and the diffusive transport properties of liquids [17,18]. They have the advantages of high ionic conductivity, easy design configuration, flexibility or self-healing [19], which provides promising ways to design high-performance and multi-functional SCs. However, despite the great progress which has been made with GPEs, their low energy density and specific capacitance are still major issues that limit their practical applications.

ILs, one typical room temperature (RT) molten salt, are used in GPEs to improve the properties of the SCs. Because IL mainly consists of organic cations and anions, it has the advantages of high conductivity, high electrochemical stability over a wide electrochemical window, non-volatility, and good thermal stability [20,21,22]. It is the attractive candidate or “green electrolytes” used in energy storage devices. Recently, the development of non-volatile, high-performance GPEs, referred to as ion gels, with various copolymer gelators, including block [23,24,25], random [26,27] and star copolymers have been extensively reported [28]. Among them, 1-butyl-3-methylimidazole tetrafluoroborate (BMIMBF_4_) has been added into GPE due to its high conductivity, low viscosity, and easy synthesis [18,29], which can effectively improve the characteristics of the solid-state polymer supercapacitor (SSC). For instance, Wang et al. prepared several GPEs based on a copolymer matrix poly-(methoxy/hexadecyl-poly(ethylene glycol) methacrylate) (PMH) and BMIMBF_4_ by solution casting method [30]. The GPEs with 60 wt% BMIMBF_4_ had good thermal stability, excellent mechanical properties, good compatibility with metallic lithium and high ionic conductivity. Jeong et al. used spray coating technology to prepare reduced graphene oxide/acid-treated, single-walled carbon nanotubes (rGO/ASWCNTs) composite electrode. The flexible supercapacitor was fabricationed using polyvinyl alcohol/1-butyl-3-methylimidazolium tetrafluoroborate (PVA/BMIMBF_4_) as GPE [20]. The specific capacitance of the flexible supercapacitor (unbent) was 62.4 F/g, decreasing to 55.7 F/g (capacitance retention rate was 89.3%) after 1000 cycles. The capacitor retained 64.6% of its initial capacitance after 300 bending and 4000 GCD cycles. Adding IL to GPE improves the ionic conductivity and specific capacitance of SSC. Although some studies have reported the symmetrical supercapacitors based on IL GPE and their electrochemical performance, most of them used inorganic active carbon materials or metal oxides as electrodes.

As is well known, carbon materials, conducting polymers, and transition metal oxides are the three categories of the electrode materials of the supercapacitor. Using inorganic active carbon (AC) electrodes is beneficial to the high power density and long cycle life of SCs [31], however, the fabrication process is complex. The preparation of active carbon SCs requires an adhesive, and the assembled layers tend to be non-uniform. Additionally, the low energy density of AC limits its application. Thus, the fabrication of SSC using conductive polymers as the electrode and GPE as the electrolyte is of particular interest. Conductive polymers, including polyaniline, polypyrrole, and polythiophene, have been studied extensively as SCs’ electrode materials due to their rapid doping/dedoping processes and good intrinsic auto-conductivity [32]. Among them, poly(3,4-ethylenedioxythiophene) (PEDOT) attracts great attention for its high conductivity [33,34], good chemical stability, and high operating potential. However, its lower power density and the capacity fading during the continuous charge/discharge processes limits its application. Therefore, in recent years, the electrochemical properties of SCs based on PEDOT have been studied and improved by modifying electrode materials, designing electrolytes or developing flexible or functional SCs to improve the electrochemical performances [35,36,37].

In this study, PEDOT-based SSCs were assembled using poly(3,4-ethylenedioxythiophene)/carbon paper (PEDOT/CP) as symmetrical electrodes and ionil liquid/poly(vinyl alchohol)/sulphuric acid (IL/PVA/H_2_SO_4_) as a GPE. Doping BMIMBF_4_ in PVA/H_2_SO_4_ GPE is expected to improve the pseudocapacitance and energy density of the SSC. The symmetric PEDOT-based SC-IL/PVA/H_2_SO_4_ has better electrical conductivity and a wider potential window than SC-PVA/H_2_SO_4_. The IL GPE was treated with freezing–thawing (F/T) cycles to improve the electrochemical performance. The effect of F/T cycles on the performances of SSC was studied using cyclic voltammetry (CV), galvanostatic charge–discharge (GCD) testing, and electrochemical impedance spectroscopy (EIS). The PEDOT SSC exhibited high energy and power densities (170.90 and 21.27 kW/kg, respectively), as well as a specific capacitance of 86.81 F/g at the discharge current of 1 mA/cm^2^. Furthermore, the IL GPE has excellent elastic recovery, mechanical properties and self-healing ability. It has great potential applications in small, flexible, and smart energy-storage devices.

## 2. Experimental

### 2.1. Materials

All chemicals were analytical grade. 3,4-ethylenedioxythiophene (EDOT, 99%) and lithium perchlorate (LiClO_4_·3H_2_O) were purchased from Aladdin Reagent Co., Ltd. Shanghai, China). H_2_SO_4_ was purchased from Chengdu Kelong chemical reagen, Chengdu, China. PVA-1799 was purchased from Shanxi Sanwei Chemical Co. Ltd., hongtong, China. BMIMBF_4_ was received from Lanzhou institute of chemical physics, Lanzhou, China. Ethanol absolute was obtained from Tianjin Damao chemical reagent factory, Tianjin, China. Carbon paper (CP, Toray 060 type) was provided by Shanghai Hesen Electric Co. Ltd., Shanghai, China.

### 2.2. Preparation of PEDOT/CP Electrode Material

CP substrate (2 × 1 cm) was soaked in ethanol absolute and deionized water, respectively, followed by ultrasonic treatment 30 min before electrodeposition to remove the impurities on the surface of CP. After that, 0.01 M EDOT monomer was added to 0.1 M LiClO_4_·3H_2_O electrolyte, stirring for 60 min. The three electrodes were inserted into the above solution, with pretreated CP as the working electrode, platinum plate (Pt) as the counter electrode, and saturated calomel electrode (SCE) as the reference electrode. PEDOT film was coated on CP by unipolar pulse electro-deposition [38]. Finally, the PEDOT/CP electrode was rinsed several times with deionized water and dried at 50 °C for 24 h. Parameters of the unipolar pulse electro-deposition were set as: voltage value: 1.1 V, on time: 0.1 s, off time: 1.0 s, pulse time: 700 times. The mass of PEDOT active materials on CP substrate is ~0.8–1.0 mg cm^−2^, which was measured with an AL104 electronic balance (Shanghai Mettler Toledo Instrument Co. Ltd., Shanghai, China).

### 2.3. Fabrication of Gel Polymer Electrolytes and Solid-State Supercapacitors

IL/PVA/H_2_SO_4_ electrolyte was prepared by the following method [39]: H_2_SO_4_ (2 g) was added to deionized water (20 mL) with magnetic stirring, then PVA (2 g) was added, stirring for 1 h at 85 °C to form a homogeneous, clear and viscous solution. Subsequently, 50 wt% BMIMBF_4_ (relative to the mass of PVA) was added, stirring for 15 min. After cooling to RT (20 °C), it was frozen at −20 °C for 10 min. A pipette was used to evenly distribute IL/PVA/H_2_SO_4_ GPE on one side of the PEDOT/CP electrodes, After 10 min, the SSC were assembled by facing two symmetrical PEDOT/CP electrodes distributed with GPE, followed by freezing at −20 °C and thawing at RT for 24 h with different numbers of F/T cycles, recorded as F/T-n (n = 1, 2, 3, 4). PEDOT SSC based on gel electrolyte without BMIMBF_4,_ i.e., PVA/H_2_SO_4_, was fabricated using the same method for comparison.

### 2.4. Electrochemical Performance Test

The electrochemical tests of PEDOT SSC were performed on multi-channel VMP3 electrochemical workstation controlled by EC-Lab software (Princeton Applied Research, Princeton, NJ, USA). The mass of PEDOT films were measured by AL104 electronic balance (Shanghai Mettler Toledo Instrument Co., Ltd., Shanghai, China). The electrochemical performances of SSC were evaluated by CV, GCD, and EIS test in ambient condition. The mass specific capacitance (C_s_) value of SSC was calculated from charge/discharge curves, according to Equation (1)

(1) $$C_{s}=\frac{I\times t_{d}}{\Delta V\times m}$$

where, *I* (A) is the discharge current, *m* (g) is the mass of electroactive materials, *t_d_* (s) is the discharge time and Δ*V* (V) represents the operating voltage window of the device.

Energy density (E, Wh/kg), power density (P, kW/kg) were calculated from the following equations

(2) $$E=\frac{1}{2}C_{s}\Delta V^{2}$$

(3) $$P=\frac{E}{t_{d}}$$

The electrical conductivity of the IL/PVA/H_2_SO_4_ GPE films was measured at RT by the SZT-2A four-point probes resistivity measurement system (Suzhou Tongchuang Electronics Co. Ltd., Suzhou, China). For each sample, at least five replicates were tested, and the results were presented as the average.

### 2.5. Differential Scanning Calorimetry (DSC)

DSC analysis was conducted on a NETZSCH DSC 204 (Selb, German) under a nitrogen atmosphere. IL/PVA/H_2_SO_4_ GPE were dried at 80 °C under vacuum oven; small pieces were then sliced for the DSC tests. Measurements were carried out from -60 to 290 °C at a heating rate of 10 °C/min.

## 3. Results and Discussion

### 3.1. Macrographs and SEM images of CP and PEDOT/CP

**Figure 1:**
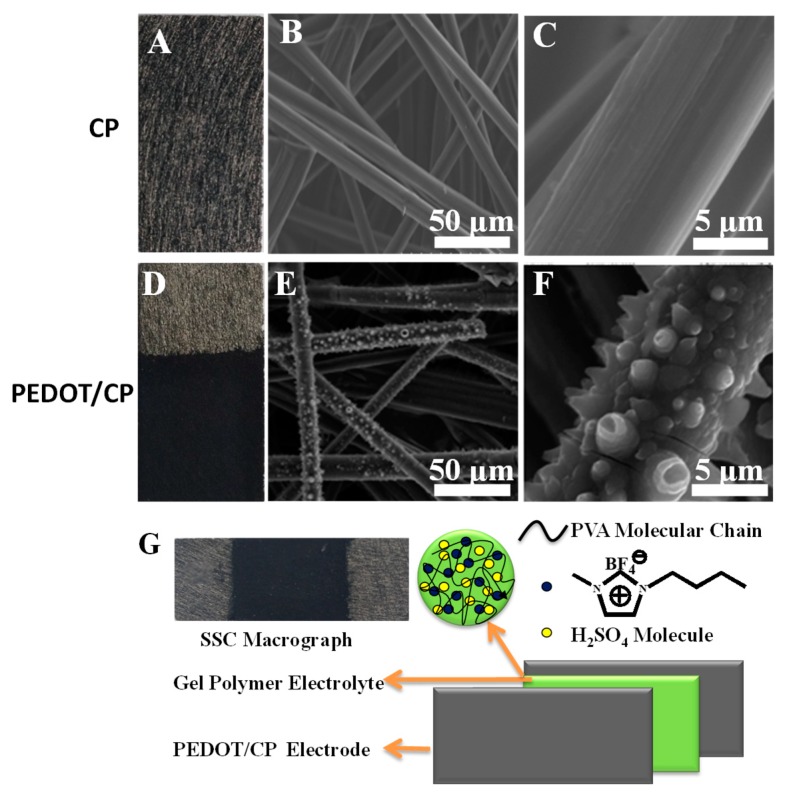
Macrographs and SEM images of the carbon paper (**A**–**C**) and PEDOT/CP (**D**–**F**), and the illustration of PEDOT solid-state polymer supercapacitor (**G**).

Figure 1A,D show macrographs of the CP and the PEDOT/CP electrode. The detail preparation of PEDOT/CP electrode was stated in our previous work [38]. The long and smooth fibers of CP orient randomly and are densely packed, forming the porous structure (Figure 1B,C) [3,40], which is help for the coating of PEDOT and provides the channel for the ion migration. Figure 1E,F shows that the CP coated with PEDOT films (PEDOT/CP) retained the loose, porous microstructure of electrode and that there are lots of spindle-like multi-sized PEDOT distributing uniformly over individual carbon fibers. In 0.5 M H_2_SO_4_ liquid electrolyte, the prepared PEDOT/CP electrode presents a high specific capacitance of 126.24 F/g at 1 mA/cm^2^ and good cycling stability, with 80.57% retention of its initial capacitance over 5000 cycles. Thus, we choose the PEDOT/CP electrode to assemble SSC using GPE and study its electrochemical properties. As illustrated in the macroscopical digital images and the illustration in Figure 1G, the two GPE-coated electrodes were assembled face to face into SSC. Its electrochemical performances were studied using CV, GCD, and EIS in the following.

### 3.2. The Cyclic Voltammetry Test of SSC Based on Different Electrolytes

**Figure 2:**
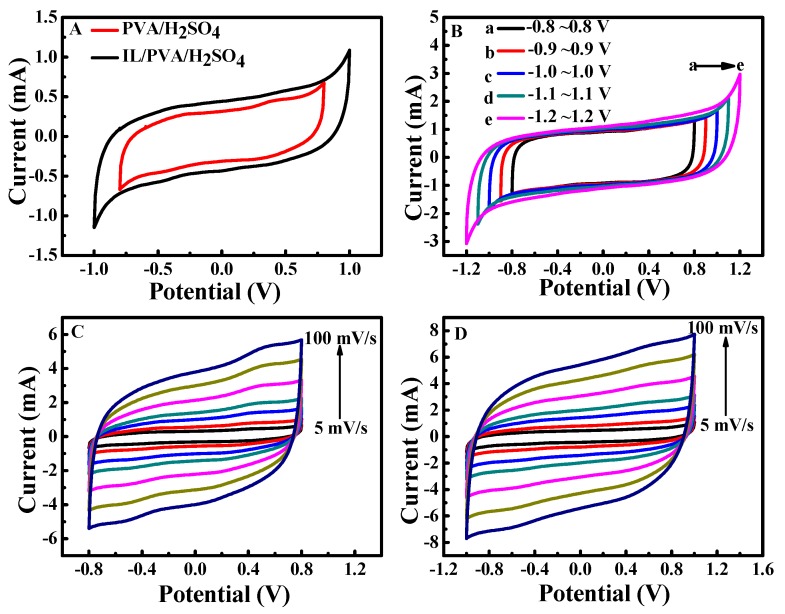
Cyclic voltammetry (CV) curves of solid-state polymer supercapacitor (SSC) based on different gel polymer electrolyte (GPE) at 5 mV/s (**A**), SSC-IL/PVA/H_2_SO_4_ at 20 mV/s within different electrochemical windows (**B**), SSC-PVA/H_2_SO_4_ (**C**) and SSC-IL/PVA/H_2_SO_4_ (**D**) at different scan rates.

Figure 2A shows the CV curves of SSC based on two different electrolytes at 5 mV/s. The rectangular shapes of the CV curves indicate their excellent capacitive behaviors. The SSC-IL/PVA/H_2_SO_4_ has larger CV area than SSC-PVA/H_2_SO_4_, which indicates that embedding BMIMBF_4_ to PVA/H_2_SO_4_ can effectively improve the capacitive performance of the device. Figure 2B shows that the CV area gradually increases when the voltage window expands from 1.6 V (−0.8~0.8 V) to 2.0 V (−1.0~1.0 V); the shape of all CV curves are close to rectangular. However, there are distinct and sharp peaks at the end of the CV curves at a maximum operating voltage of 1.2 V; this indicates that higher electrochemical potential will cause the decomposition of water [17,41,42,43]. Thus, we choose −1.0–1.0 V as the optimal operating voltage window for SSC. The rectangular shape is generally retained even when the scan rate is increased to 100 mV/s, which indicates the good rate performance of SSC.

### 3.3. The Galvanostatic Charge–Discharge Test

**Figure 3:**
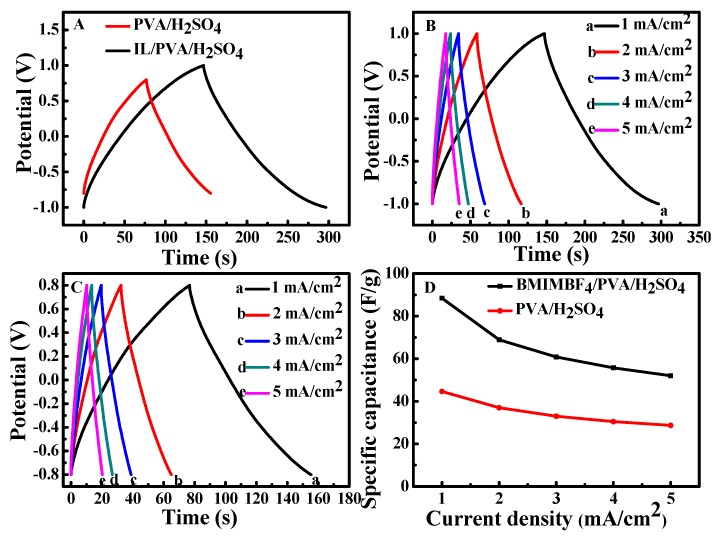
Galvanostatic charge–discharge (GCD) curves of the SSC based on different GPEs at 1 mA/cm^2^ (**A**), GCD curves of SSC-IL/ PVA/H_2_SO_4_ (**B**) and SSC-PVA/H_2_SO_4_ (**C**), as well as the specific capacitances (**D**).

GCD tests were carried out to further evaluate the performance of SSC based on the two kinds of electrolytes, and the results are shown in Figure 3. It can be seen that the discharging time of SSC-IL/PVA/H_2_SO_4_ is obviously longer than that of SSC-PVA/H_2_SO_4_, indicating the former has a better supercapacitive performance [14]. The IL GPE can provide electro-active ions (BMIM^+^ and BF_4_^−^) for the electrode and electrolyte interface, thereby enhancing specific capacitance [20]. The GCD curves in Figure 3B,C indicate that the charge–discharge time is approximately equal and the coulombic efficiency is close to 100%, which means the two SSCs have good electrochemical reversibility [9,44,45]. The specific capacitances calculated from the GCD curves according to Equation (1) are plotted as a function of the discharge current density (Figure 3D). Compared with the SSC-PVA/H_2_SO_4_, the SSC-IL/PVA/H_2_SO_4_ has better electrochemical behavior; the specific capacitance increases by two times with the introduction of IL. The capacitance of SSC-IL/PVA/H_2_SO_4_ decreases from 86.81 to 52.15 F/g with the gradual increase in current density from 1 to 5 mA/cm^2^. The slight fading of the capacitance up to a high current density is attributed to the fast redox at the electrode/electrolyte interfaces.

### 3.4. The Electrochemical Impedance Spectroscopy Test

**Figure 4:**
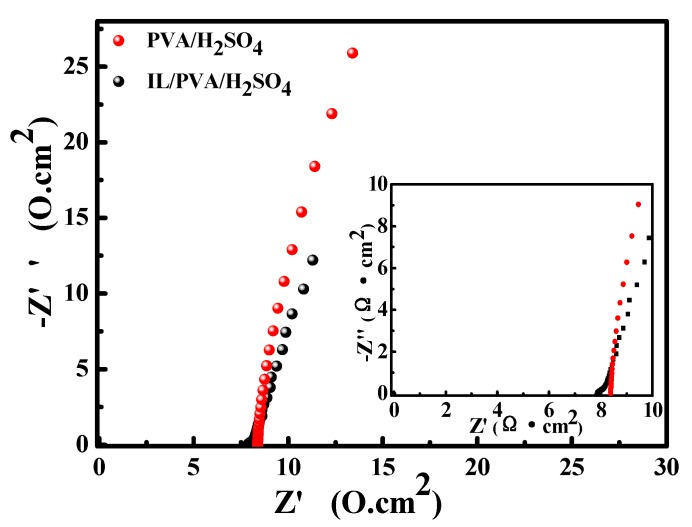
Electrochemical impedance spectra of the SSC based on IL/PVA/H_2_SO_4_ and PVA/H_2_SO_4_ GPEs and a magnified view of the high-frequency region.

The EIS results of the two SSCs are shown in Figure 4, and the corresponding impedance fitting data are listed in Table 1. R_s_ is the internal resistance related to the ionic resistance of the electrolyte, and the contact resistance at the interface of the electrode/electrolyte. R_ct_ is the charge transfer resistance occurring at the electrode/electrolyte interface during the charging–discharging process. The R_s_ and R_ct_ values are calculated from the results EIS. Compared with SSC-PVA/H_2_SO_4_, the R_s_ and R_ct_ of SSC-IL/PVA/H_2_SO_4_ is relatively smaller because IL can be used as an ion source to obtain more carriers in IL-PVA/H_2_SO_4_, including imidazole cations BMIM^+^ and BF_4_^−^ apart from H^+^ and SO4^2−^ [14]. The four-point probe method result also confirms that the conductivity of IL/PVA/H_2_SO_4_ (90.09 mS/cm) polymer gel is better than that of PVA/H_2_SO_4_ (38.36 mS/cm). With the recent great progress in polymer gel, some work focuses on how to improve its conductivity. Peng [46] reported the graphite oxide (GO) doped PVA/KCl/GO hydrogel had a high ionic conductivity of 47.5 mS/cm (2.3 wt% GO) compared with bare B-PVA/KCl hydrogel (32.6 mS/cm). Ramesh group [47] showed the ionic conductivity of solid polymer electrolytes based on PVA incorporated with sodium salt and ionic liquid improved the conductivity from 4.87 × 10^−3^ to 2.31 mS/cm. Hashaikeh and coworkers [48] showed the sulfated cellulose/PVA composites as proton-conducting electrolyte for capacitors activated with 0.25M H_2_SO_4_ show a high ionic conductivity of 250 mS/cm. These studies suggest that the introduction of IL or H_2_SO_4_ has a great contribution to the ionic conductivity of the PVA-based gel. Therefore, the high conductivity of SSC-IL/PVA/H_2_SO_4_ can be attributed to the coefficient of all ions, including BMIM^+^, BF^4−^, H^+^ and SO_4_^2−^.

### 3.5. The Energy Density and Power Density Test

**Figure 5:**
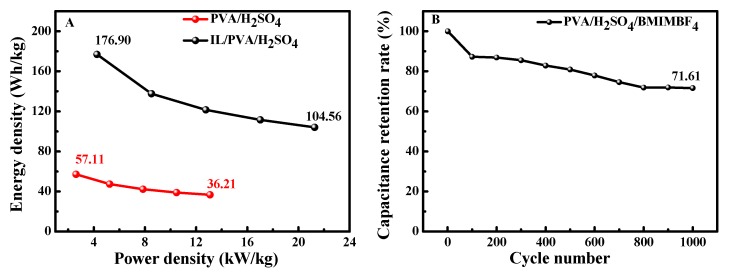
Ragone plot of the SSC based on two GPEs (**A**) and the capacitance retention rate of SSC-IL/PVA/H_2_SO_4_ (**B**) at 5 mA/cm^2.^

Energy density (E) is an important influence parameter for SSC in practice. The E and power density (P) of SSC based on the two electrolytes are evaluated and the results are shown in Figure 5A. The SSC-IL/PVA/H_2_SO_4_ shows a much higher E and P than SSC-PVA/H_2_SO_4_. The maximum P is 21.27 kW/kg at a current density of 5 mA/cm^2^, and the optimal energy density is up to 176.90 Wh/kg at 1 mA/cm^2^, which is about 3.1 times higher than that of SSC-PVA/H_2_SO_4_ (57.11 Wh/kg). This indicates that adding BMIMBF_4_ to PVA/H_2_SO_4_ electrolyte can improve the capacitive behavior. The values of E and P are comparable to many recently reported PEDOT electrodes-based SCs. For example, Pandey and coworkers studied the electrochemical energy storage performance of SCs fabricated using PEDOT-coated carbon fiber paper electrodes and IL GPE in acetone [13]. It had a maximum specific capacitance of 154.4 F/g and the specific power and energy values were found to be 11.3 and 6.5 Wh/kg, respectively. Compared to this, the specific power and specific energy in this work have been improved. Moreover, we use aqueous IL-GPE rather than organic solvent base IL-GPE; the assembled SSC-IL/PVA/H_2_SO_4_ is nontoxic, green or eco-friendly, and more promising. The cycling stability is one of the most important parameters for the supercapacitor. As Figure 5B shows, the capacitive retention rate of SSC-IL/PVA/H_2_SO_4_ decreases to 71.61% after 1000 cycles. The capacitance fading is mainly attributed to the slightly decreased electroactivity of PEDOT.

### 3.6. The Effect of Freezing–Thawing Cycles on the Electrochemical Properties of SSC

Due to the regularly arranged hydroxyl groups along the PVA chains, PVA hydrogels are commonly prepared through the strong physical crosslinking points generated by the repeated F/T method [18,49]. The physical crosslinking is constructed through the crystallite region and the hydrogen bond inter- or intro-PVA chains. Therefore, it is expected that the two conducting polymer electrodes can be assembled as SSCs using a PVA-based ionic liquid gel as the electrolyte. The number of F/T cycles is one important condition that must be taken into account for the structure and properties of PVA hydrogel. A higher number of F/T cycles will increase the physically crosslinked PVA macromolecules, which are bound into a network, thus improving the stability and elastic properties of the hydrogel [18,50,51]. To obtain the excellent properties, herein we study the effect of the number of F/T cycles on the SSC.

**Figure 6:**
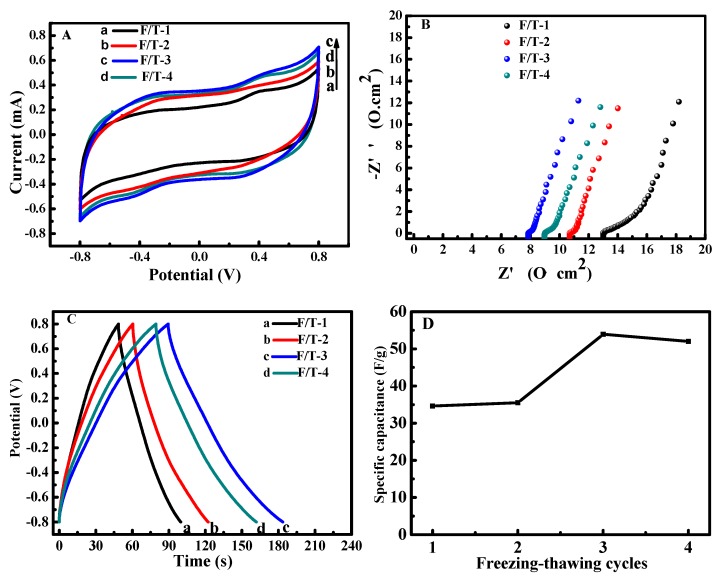
Cyclic voltammetry curves (**A**), electrochemical impedance spectra (**B**) of SSC-IL/PVA/H_2_SO_4_ with various freezing-thawing cycles at 5 mV/s, galvanic charge–discharge curves (**C**), and the specific capacitance (**D**) at 1 mA/cm^2^.

Figure 6A shows that the CV curves of SSC-IL/PVA/H_2_SO_4_ applied with different F/T cycles have quasi-rectangular shapes. The CV area and the response current slightly increase firstly and then decrease with the higher F/T cycles. The F/T-3 has the optimum performance. The Nyquist curves in Figure 6B show that the internal resistance of SSC depends on the number of F/T cycles. The device subjected to three F/T cycles has the minimum internal resistance, as listed in Table 2, because the stable network of GPE with three F/T cycles can provide effective channels for the ions’ transportation. The corresponding R_ct_ is relatively large, which is due to the formation of ion layers on the electrode surface during the embedding/embedding process, resulting in the increasing impedance [52]. However, too many physical crosslinking points will generate in the GPE when the F/T is more than three cycles, which will hinder the ion transportation. Figure 6C shows that all GCD curves display good symmetry, indicating that the SSCs have excellent electrochemical reversibility. In addition, the nearly linear discharge behavior exhibits the good capacitive performance of SSCs. The charge–discharge time and specific capacitance gradually increase, and then decrease with the F/T cycles, reaching a maximum value of 53.73 F/g at three F/T cycles.

### 3.7. The Effect of Freezing–Thawing Cycles on the Thermal Properties of SSC-IL/ PVA/H_2_SO_4_

**Figure 7:**
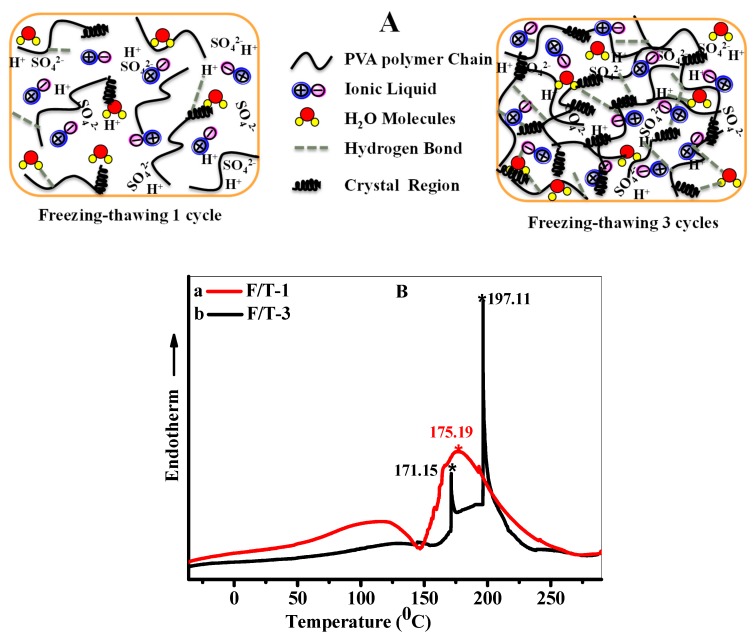
The structure illustration (**A**) and DSC thermogram (**B**) of IL/PVA/H_2_SO_4_ GPE subjected to different freezing–thawing cycles.

The intermolecular interaction increases between pendant hydroxyl groups, forming a crystallite region, and generating a three-dimensional polymer gel network, where the crystallites and the polymer chains entanglement act as knots [53,54], while the pores are filled by the ionic liquid, H_2_SO_4_ and free water. The polymer network is not stable or perfect when the SSC is subjected one F/T cycle. Upon increasing the number of F/T cycles to three, the numbers of hydrogen bond in the gel increases, and the physical cross-linking points also increase due to the polymer chains’ entanglement, forming a gel with stable crosslinking network as shown in Figure 7A [55], which allows ionics to transfer in the electrolyte. A higher degree of crystallinity, present in the PVA hydrogen with higher F/T cycles, is confirmed by DSC thermograms of IL/PVA/H_2_SO_4_ GPE subjected to one or three F/T cycles. For sample F/T-1, the peak observed at 100 °C is considered as a chain movement caused by water or acid, IL molecules release or movement. The peak at 175.19 °C is the melting temperature caused by the limited crystal region. For sample F/T-3, the peak present at 171.15 °C becomes weak and the main melting peak shifts to a higher temperature, implying that the increasing degree of crystallization is due to the increasing F/T cycles.

### 3.8. The Mechanical Properties and Function of Ionic Liquid-Based Polymer Gel

**Figure 8:**
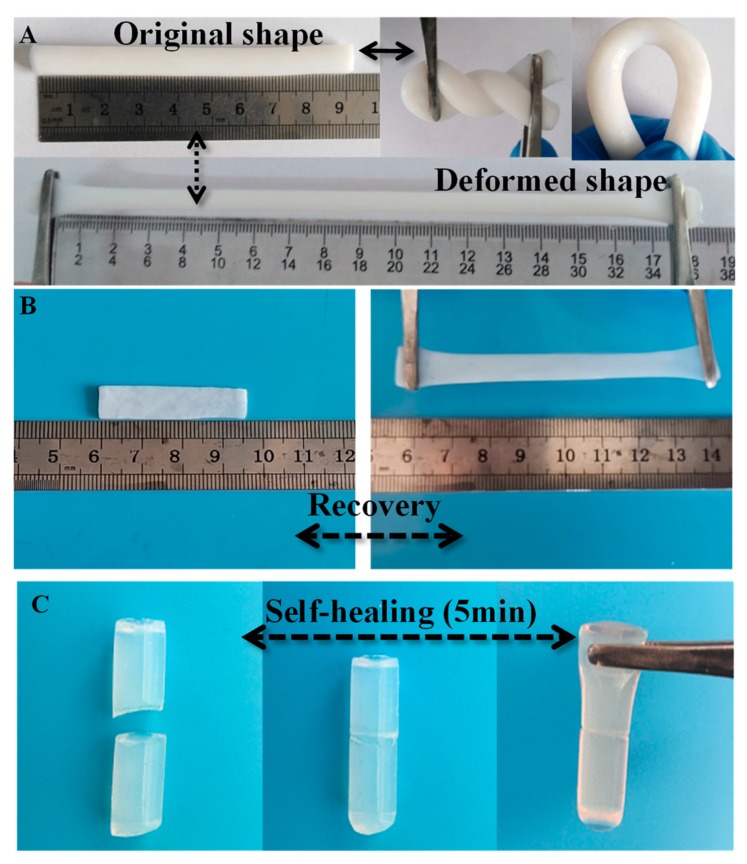
The digital photography of the elastic deformation, recovery (**A**,**B**) and the self-healing (**C**) of IL/PVA/H_2_SO_4_ polymer gel.

The GPE was sandwiched between the two PEDOT/CP electrodes. It is not convenient to study the appearance, elasticity recovery, mechanical properties or other functional characteristics, so we fabricated the film and rod shape of the GPE sample using the same preparation method to directly observe the mechanical properties. The mechanical property of the GPE is the critical parameter that will affect the applications [56]. Figure 8A,B shows the IL/PVA/H_2_SO_4_ gel film or road sample is soft and flexible, and it has excellent recovery elasticity. It can be easily stretched at least two times or twist under extra force and fully recover to its original shape just like rubber, indicating that GPE has excellent mechanical properties. What is interesting is the IL GPE has excellent self-healing properties, as shown in Figure 8C; the road sample was cut in the middle and it can recover to its original state after 5 min in air at RT, and we can pick up the self-healed sample with tweezers. The self-healing can be attributed to the hydrogen bonding interaction during the formation of the IL polymer gel. Although the hydrogel electrolytes have made great progress, they rarely present self-healing behavior [46]. Furthermore, it still faces great challenges to assemble the flexible SCs with good specific capacity, long life cycles, high ionic conductivity and excellent self-healing when suffering damage [57]. This will open a window for studying the flexible and self-healing smart supercapacitor or other multi-functional electrical devices. Future work will focus on this and will be carried out continuously in this field.

## 4. Conclusions

In this paper, the symmetrical SSC was fabricated using PEDOT/CP prepared by the unipolar pulse method as electrodes, GPE based on IL/PVA/H_2_SO_4_ as GPE. IL/PVA/H_2_SO_4_ GPEs were prepared by F/T cycle. The electrochemical performances of SSC were tested by CV, GCD, and EIS. Compared with the SSC-PVA/H_2_SO_4_, the mass specific capacitance of SSC-IL/PVA/H_2_SO_4_ was improved by two times, reaching 86.81 F/g. And its energy density was also significantly improved to reach 176.90 Wh/kg. In addition, the SSC-IL/PVA/H_2_SO_4_ had good cycle stability, which retained 71.61% of the initial capacitance after 1000 GCD cycles. Therefore, adding IL (BMIMBF_4_) to PVA/H_2_SO_4_ GPE can effectively improve the capacitive performance and energy density of SSC. Furthermore, IL/PVA/H_2_SO_4_ GPE also exhibits good elastic recovery and self-healing, which gives it significant application value in light, thin and wearable flexible devices due to its excellent mechanical properties.

## Figures and Tables

**Table 1:** Resistance values of the SSC based on IL/PVA/H_2_SO_4_ and PVA/H_2_SO_4._

Electrolyte	R_s_ (Ω·cm^2^)	R_ct_ (Ω·cm^2^)	Conductivity (mS/cm)
PVA/H_2_SO_4_	8.38	0.010	38.36
IL/PVA/H_2_SO_4_	7.90	2.608 × 10^−3^	90.09

R_s_: Resistive properties of the electrolyte and electrode. R_ct_: The interfacial charge transfer resistance of electroactive materials.

**Table 2:** Resistance values of SSC-IL/PVA/H_2_SO_4_ underwent different freezing–thawing (F/T) cycles.

F/T Cycles	R_s_ (Ω·cm^2^)	R_ct_ (Ω·cm^2^)	C_p_ (F/g)	T_m_ (°C)	Conductivity (mS/cm)
1	13.00	1.013 × 10^−7^	34.47	175.19	68.81
3	7.90	2.608 × 10^−3^	53.73	197.11	90.09

R_s_: Resistive properties of the electrolyte and electrode. R_ct_: The interfacial charge transfer resistance of electroactive materials. R_s_ and R_ct_ were determined from the results of EIS. T_m_: Melting Temperature F/T: Freezing–thawing. T_m_ is from the DSC result, and Conductivity is from the four-point probe method.
